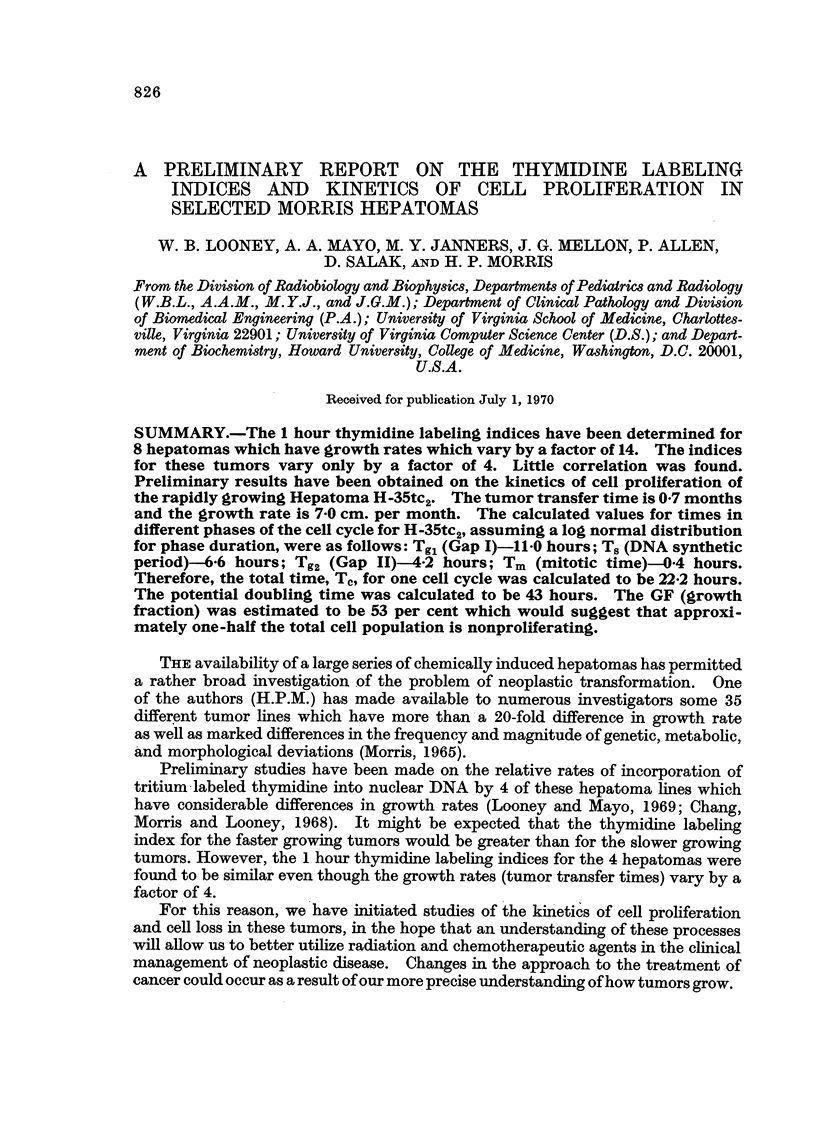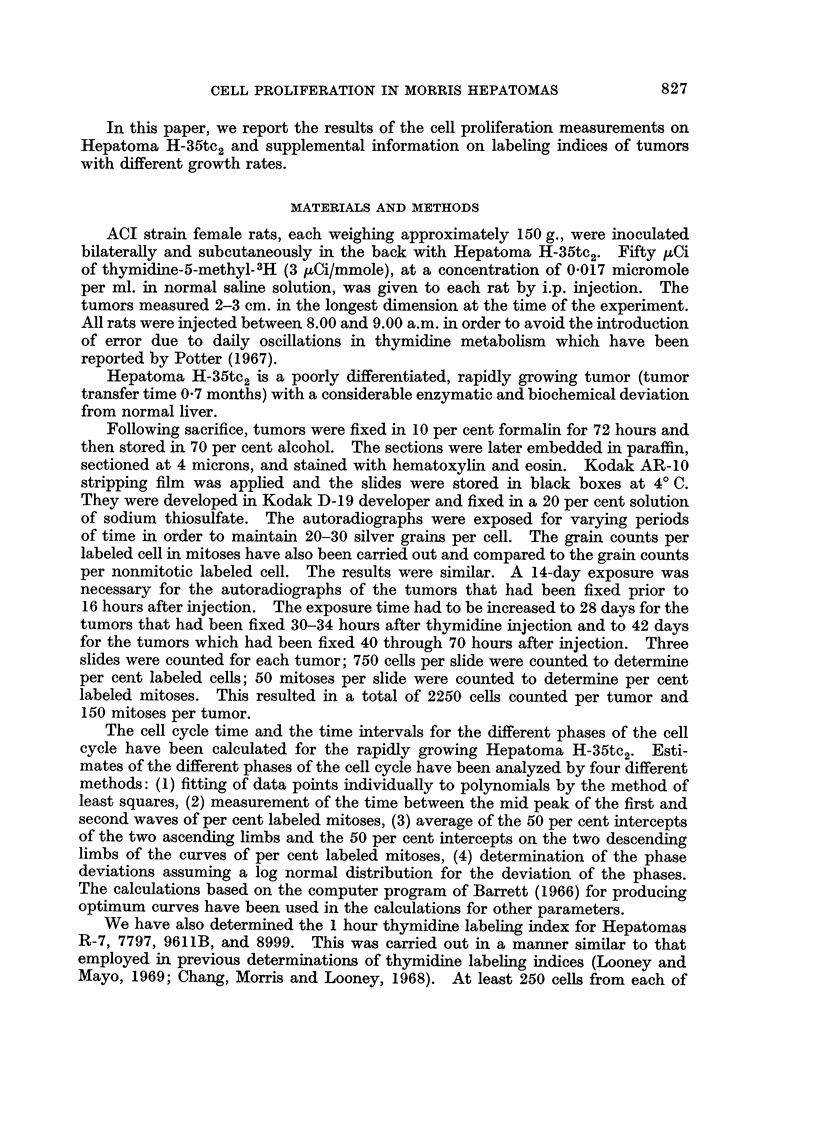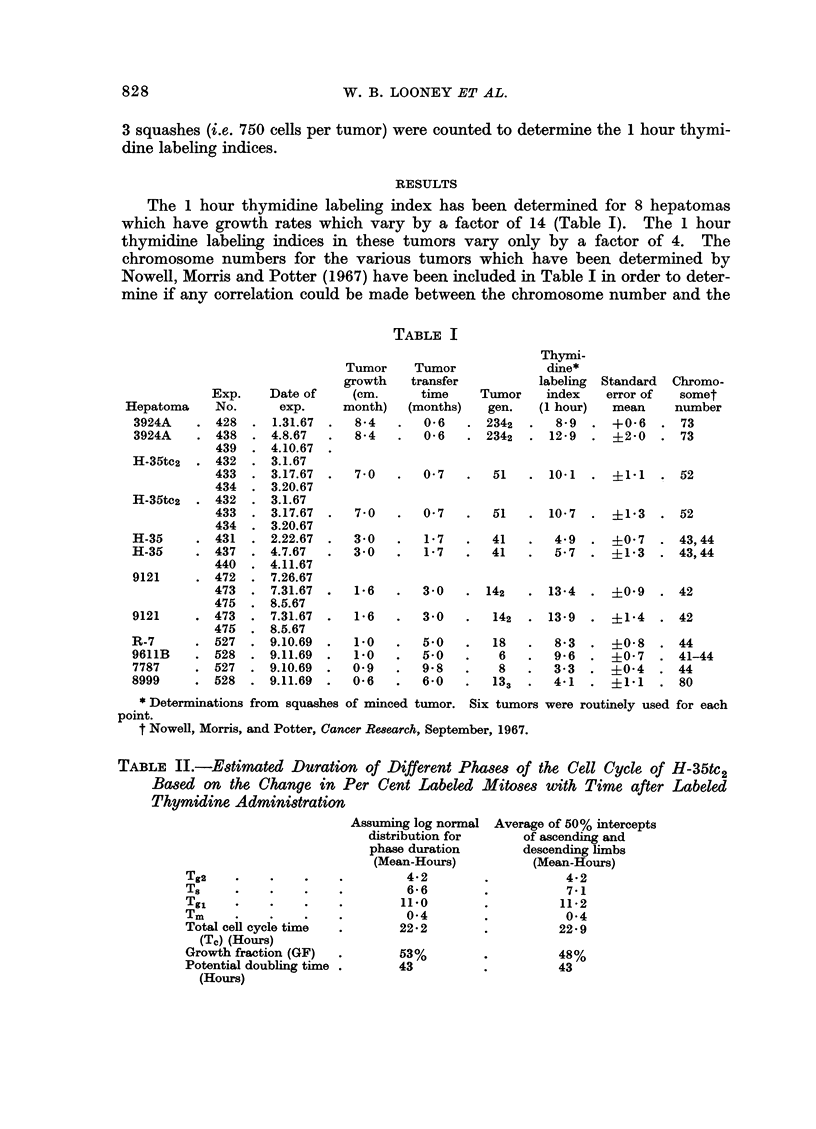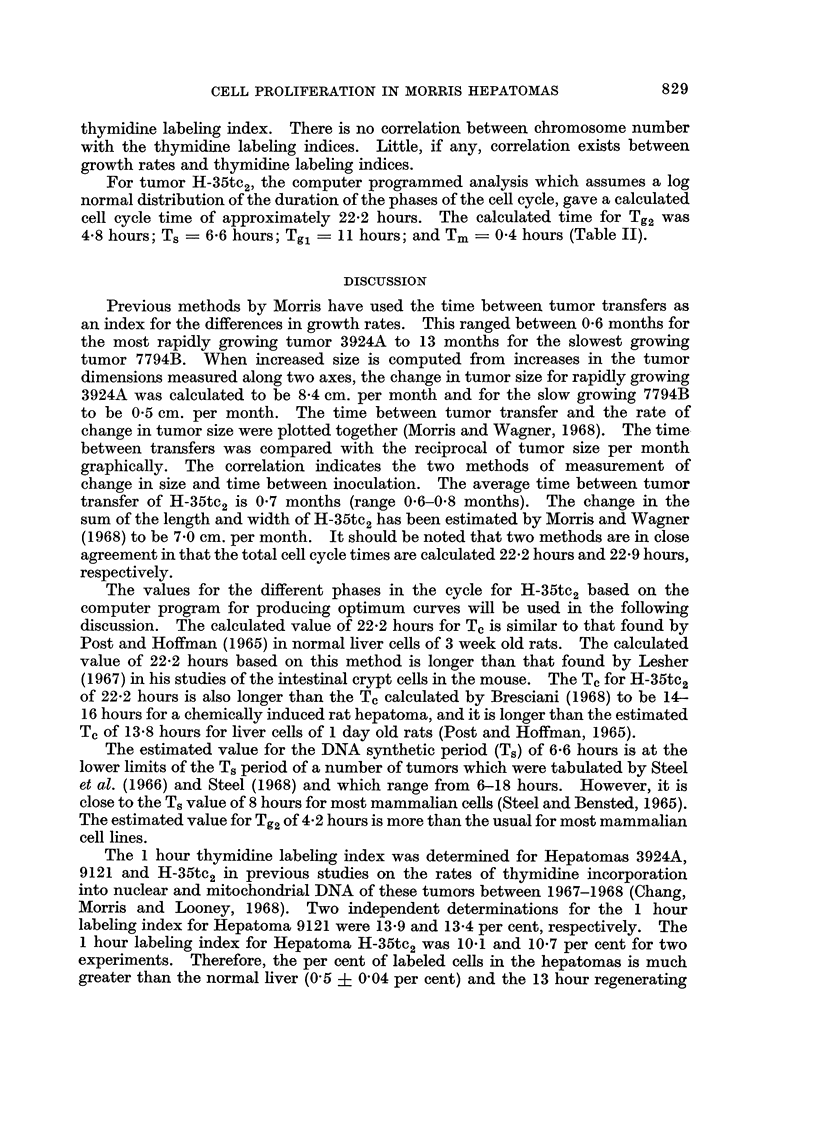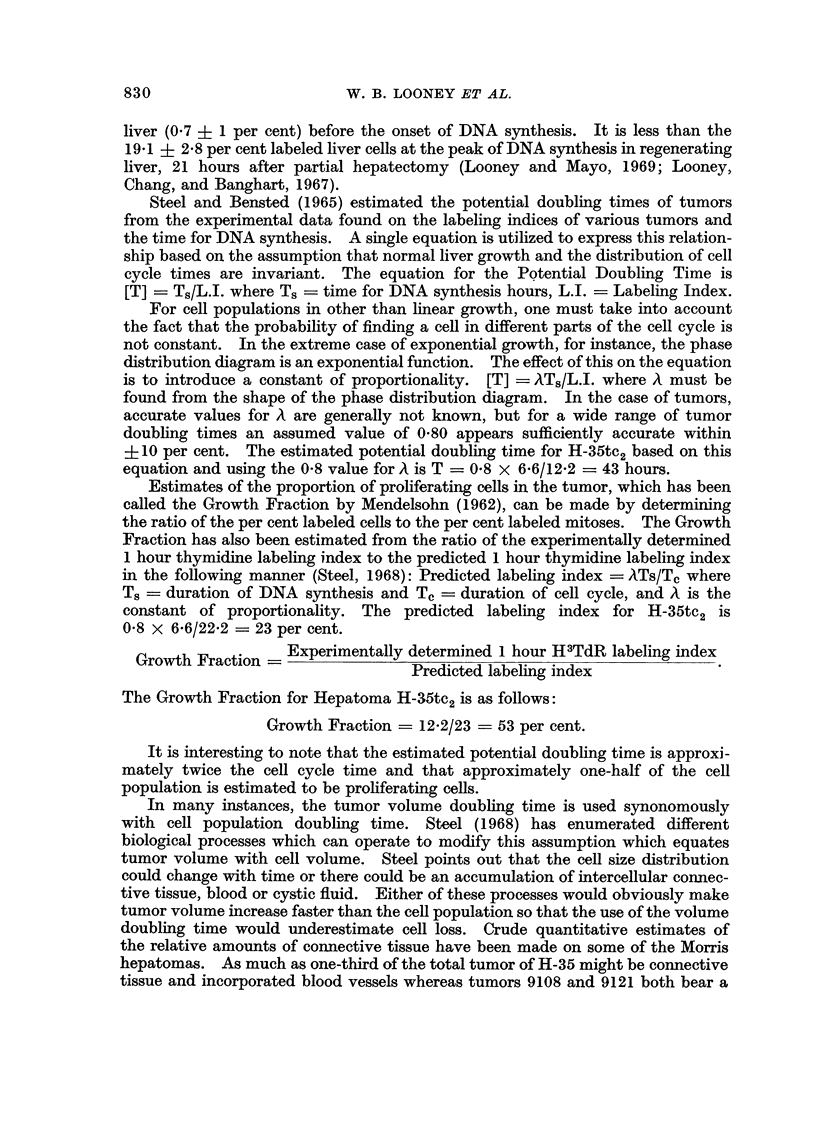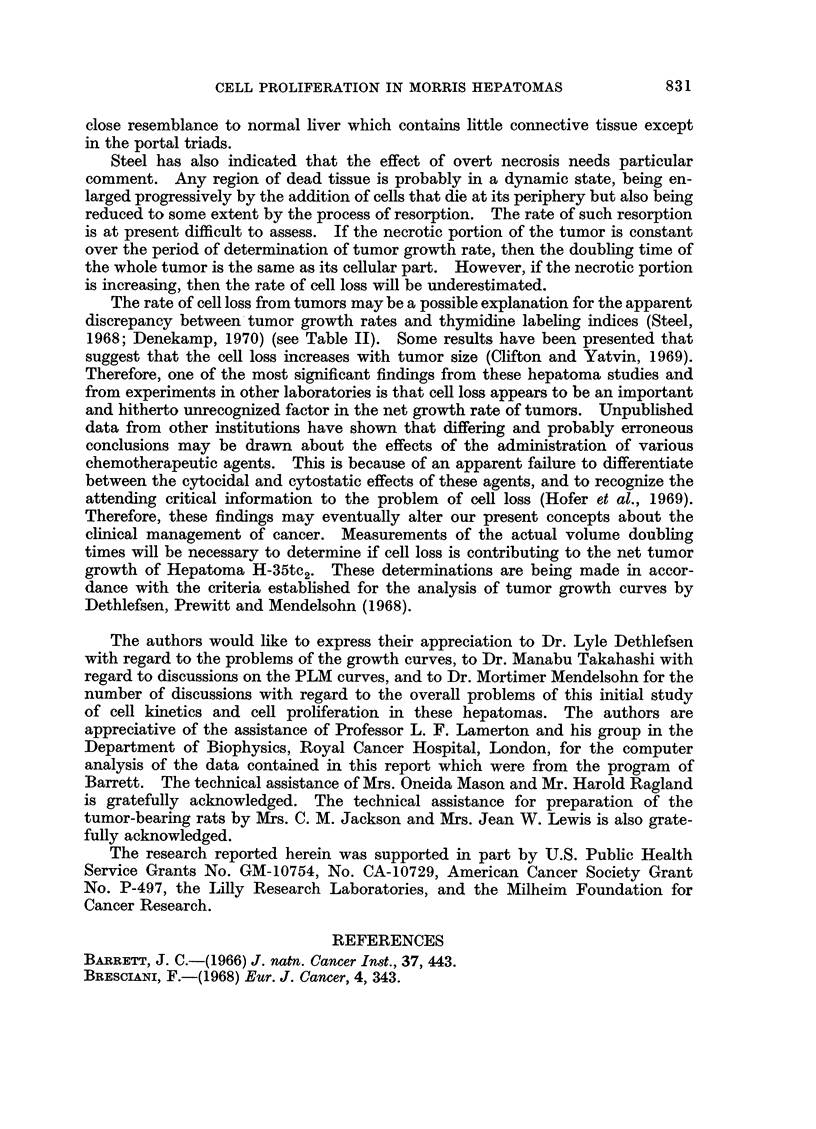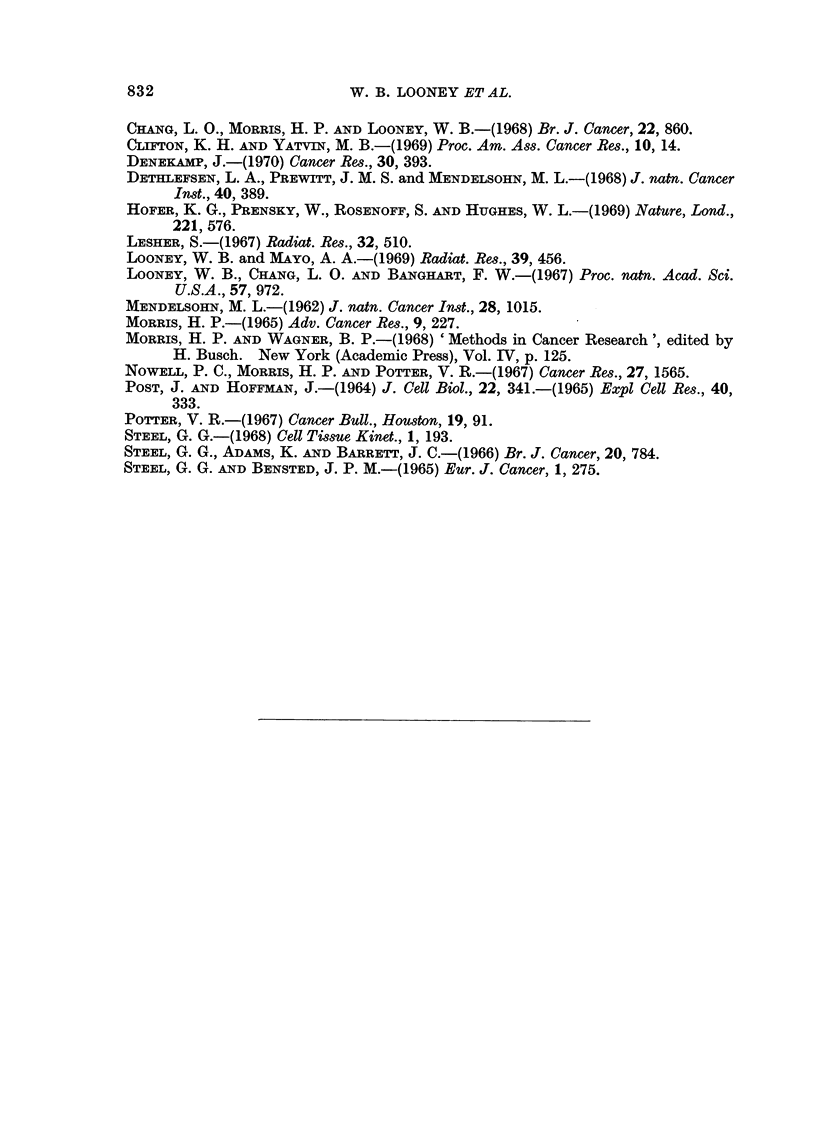# A Preliminary Report on the Thymidine Labeling Indices and Kinetics of Cell Proliferation in Selected Morris Hepatomas

**DOI:** 10.1038/bjc.1970.97

**Published:** 1970-12

**Authors:** W. B. Looney, A. A. Mayo, M. Y. Janners, J. G. Mellon, P. Allen, D. Salak, H. P. Morris

## Abstract

The 1 hour thymidine labeling indices have been determined for 8 hepatomas which have growth rates which vary by a factor of 14. The indices for these tumors vary only by a factor of 4. Little correlation was found. Preliminary results have been obtained on the kinetics of cell proliferation of the rapidly growing Hepatoma H-35tc_2_. The tumor transfer time is 0.7 months and the growth rate is 7.0 cm. per month. The calculated values for times in different phases of the cell cycle for H-35tc_2_, assuming a log normal distribution for phase duration, were as follows: T_g1_ (Gap I)—11.0 hours; T_s_ (DNA synthetic period)—6.6 hours; T_g2_ (Gap II)—4.2 hours; T_m_ (mitotic time)—0.4 hours. Therefore, the total time, T_c_, for one cell cycle was calculated to be 22.2 hours. The potential doubling time was calculated to be 43 hours. The GF (growth fraction) was estimated to be 53 per cent which would suggest that approximately one-half the total cell population is nonproliferating.


### A PRELIMINARY REPORT ON THE THYMIDINE LABELING INDICES AND KINETICS OF CELL PROLIFERATION IN SELECTED MORRIS HEPATOMAS

W. B. LOONEY, A. A. MAYO, M. Y. JANNERS, J. G. MELLON, P. ALLEN, D. SALAK, AND H. P. MORRIS

*From the Division of Radiobiology and Biophysics, Departments of Pediatrics and Radiology (W.B.L., A.A.M., M.Y.J., and J.G.M.); Department of Clinical Pathology and Division of Biomedical Engineering (P.A.); University of Virginia School of Medicine, Charlottesville, Virginia* 22901; *University of Virginia Computer Science Center (D.S.); and Department of Biochemistry, Howard University, College of Medicine, Washington, D.C.* 20001, *U.S.A.*

Received for publication July 1, 1970

**SUMMARY.—The 1 hour thymidine labeling indices have been determined for 8 hepatomas which have growth rates which vary by a factor of 14. The indices for these tumors vary only by a factor of 4. Little correlation was found. Preliminary results have been obtained on the kinetics of cell proliferation of the rapidly growing Hepatoma H-35tc$_2$. The tumor transfer time is 0·7 months and the growth rate is 7·0 cm. per month. The calculated values for times in different phases of the cell cycle for H-35tc$_2$, assuming a log normal distribution for phase duration, were as follows: $T_{g1}$ (Gap I)—11·0 hours; $T_s$ (DNA synthetic period)—6·6 hours; $T_{g2}$ (Gap II)—4·2 hours; $T_m$ (mitotic time)—0·4 hours. Therefore, the total time, $T_c$, for one cell cycle was calculated to be 22·2 hours. The potential doubling time was calculated to be 43 hours. The GF (growth fraction) was estimated to be 53 per cent which would suggest that approximately one-half the total cell population is nonproliferating.**

THE availability of a large series of chemically induced hepatomas has permitted a rather broad investigation of the problem of neoplastic transformation. One of the authors (H.P.M.) has made available to numerous investigators some 35 different tumor lines which have more than a 20-fold difference in growth rate as well as marked differences in the frequency and magnitude of genetic, metabolic, and morphological deviations (Morris, 1965).

Preliminary studies have been made on the relative rates of incorporation of tritium labeled thymidine into nuclear DNA by 4 of these hepatoma lines which have considerable differences in growth rates (Looney and Mayo, 1969; Chang, Morris and Looney, 1968). It might be expected that the thymidine labeling index for the faster growing tumors would be greater than for the slower growing tumors. However, the 1 hour thymidine labeling indices for the 4 hepatomas were found to be similar even though the growth rates (tumor transfer times) vary by a factor of 4.

For this reason, we have initiated studies of the kinetics of cell proliferation and cell loss in these tumors, in the hope that an understanding of these processes will allow us to better utilize radiation and chemotherapeutic agents in the clinical management of neoplastic disease. Changes in the approach to the treatment of cancer could occur as a result of our more precise understanding of how tumors grow.

In this paper, we report the results of the cell proliferation measurements on Hepatoma H-35tc$_2$ and supplemental information on labeling indices of tumors with different growth rates.

### MATERIALS AND METHODS

ACI strain female rats, each weighing approximately 150 g., were inoculated bilaterally and subcutaneously in the back with Hepatoma H-35tc$_2$. Fifty $\mu$Ci of thymidine-5-methyl-$^3$H (3 $\mu$Ci/mmole), at a concentration of 0·017 micromole per ml. in normal saline solution, was given to each rat by i.p. injection. The tumors measured 2–3 cm. in the longest dimension at the time of the experiment. All rats were injected between 8.00 and 9.00 a.m. in order to avoid the introduction of error due to daily oscillations in thymidine metabolism which have been reported by Potter (1967).

Hepatoma H-35tc$_2$ is a poorly differentiated, rapidly growing tumor (tumor transfer time 0·7 months) with a considerable enzymatic and biochemical deviation from normal liver.

Following sacrifice, tumors were fixed in 10 per cent formalin for 72 hours and then stored in 70 per cent alcohol. The sections were later embedded in paraffin, sectioned at 4 microns, and stained with hematoxylin and eosin. Kodak AR-10 stripping film was applied and the slides were stored in black boxes at 4° C. They were developed in Kodak D-19 developer and fixed in a 20 per cent solution of sodium thiosulfate. The autoradiographs were exposed for varying periods of time in order to maintain 20–30 silver grains per cell. The grain counts per labeled cell in mitoses have also been carried out and compared to the grain counts per nonmitotic labeled cell. The results were similar. A 14-day exposure was necessary for the autoradiographs of the tumors that had been fixed prior to 16 hours after injection. The exposure time had to be increased to 28 days for the tumors that had been fixed 30–34 hours after thymidine injection and to 42 days for the tumors which had been fixed 40 through 70 hours after injection. Three slides were counted for each tumor; 750 cells per slide were counted to determine per cent labeled cells; 50 mitoses per slide were counted to determine per cent labeled mitoses. This resulted in a total of 2250 cells counted per tumor and 150 mitoses per tumor.

The cell cycle time and the time intervals for the different phases of the cell cycle have been calculated for the rapidly growing Hepatoma H-35tc$_2$. Estimates of the different phases of the cell cycle have been analyzed by four different methods: (1) fitting of data points individually to polynomials by the method of least squares, (2) measurement of the time between the mid peak of the first and second waves of per cent labeled mitoses, (3) average of the 50 per cent intercepts of the two ascending limbs and the 50 per cent intercepts on the two descending limbs of the curves of per cent labeled mitoses, (4) determination of the phase deviations assuming a log normal distribution for the deviation of the phases. The calculations based on the computer program of Barrett (1966) for producing optimum curves have been used in the calculations for other parameters.

We have also determined the 1 hour thymidine labeling index for Hepatomas R-7, 7797, 9611B, and 8999. This was carried out in a manner similar to that employed in previous determinations of thymidine labeling indices (Looney and Mayo, 1969; Chang, Morris and Looney, 1968). At least 250 cells from each of

3 squashes (*i.e.* 750 cells per tumor) were counted to determine the 1 hour thymidine labeling indices.

### RESULTS

The 1 hour thymidine labeling index has been determined for 8 hepatomas which have growth rates which vary by a factor of 14 (Table I). The 1 hour thymidine labeling indices in these tumors vary only by a factor of 4. The chromosome numbers for the various tumors which have been determined by Nowell, Morris and Potter (1967) have been included in Table I in order to determine if any correlation could be made between the chromosome number and the

TABLE I

| Hepatoma | Exp. No. | Date of exp. | Tumor growth (cm. month) | Tumor transfer time (months) | Tumor gen. | Thymidine* labeling index (1 hour) | Standard error of mean | Chromosome† number |
|---|---|---|---|---|---|---|---|---|
| 3924A | 428 | 1.31.67 | 8·4 | 0·6 | $234_2$ | 8·9 | +0·6 | 73 |
| 3924A | 438 | 4.8.67 | 8·4 | 0·6 | $234_2$ | 12·9 | ±2·0 | 73 |
| | 439 | 4.10.67 | | | | | | |
| H-35tc2 | 432 | 3.1.67 | | | | | | |
| | 433 | 3.17.67 | 7·0 | 0·7 | 51 | 10·1 | ±1·1 | 52 |
| | 434 | 3.20.67 | | | | | | |
| H-35tc2 | 432 | 3.1.67 | | | | | | |
| | 433 | 3.17.67 | 7·0 | 0·7 | 51 | 10·7 | ±1·3 | 52 |
| | 434 | 3.20.67 | | | | | | |
| H-35 | 431 | 2.22.67 | 3·0 | 1·7 | 41 | 4·9 | ±0·7 | 43, 44 |
| H-35 | 437 | 4.7.67 | 3·0 | 1·7 | 41 | 5·7 | ±1·3 | 43, 44 |
| | 440 | 4.11.67 | | | | | | |
| 9121 | 472 | 7.26.67 | | | | | | |
| | 473 | 7.31.67 | 1·6 | 3·0 | $14_2$ | 13·4 | ±0·9 | 42 |
| | 475 | 8.5.67 | | | | | | |
| 9121 | 473 | 7.31.67 | 1·6 | 3·0 | $14_2$ | 13·9 | ±1·4 | 42 |
| | 475 | 8.5.67 | | | | | | |
| R-7 | 527 | 9.10.69 | 1·0 | 5·0 | 18 | 8·3 | ±0·8 | 44 |
| 9611B | 528 | 9.11.69 | 1·0 | 5·0 | 6 | 9·6 | ±0·7 | 41–44 |
| 7787 | 527 | 9.10.69 | 0·9 | 9·8 | 8 | 3·3 | ±0·4 | 44 |
| 8999 | 528 | 9.11.69 | 0·6 | 6·0 | $13_3$ | 4·1 | ±1·1 | 80 |

* Determinations from squashes of minced tumor. Six tumors were routinely used for each point.

† Nowell, Morris, and Potter, *Cancer Research*, September, 1967.

TABLE II.—*Estimated Duration of Different Phases of the Cell Cycle of H-35tc$_2$ Based on the Change in Per Cent Labeled Mitoses with Time after Labeled Thymidine Administration*

| | Assuming log normal distribution for phase duration (Mean-Hours) | Average of 50% intercepts of ascending and descending limbs (Mean-Hours) |
|---|---|---|
| $T_{g2}$ | 4·2 | 4·2 |
| $T_s$ | 6·6 | 7·1 |
| $T_{g1}$ | 11·0 | 11·2 |
| $T_m$ | 0·4 | 0·4 |
| Total cell cycle time ($T_c$) (Hours) | 22·2 | 22·9 |
| Growth fraction (GF) | 53% | 48% |
| Potential doubling time (Hours) | 43 | 43 |

thymidine labeling index. There is no correlation between chromosome number with the thymidine labeling indices. Little, if any, correlation exists between growth rates and thymidine labeling indices.

For tumor H-35tc$_2$, the computer programmed analysis which assumes a log normal distribution of the duration of the phases of the cell cycle, gave a calculated cell cycle time of approximately 22·2 hours. The calculated time for $T_{g_2}$ was 4·8 hours; $T_s = 6{\cdot}6$ hours; $T_{g_1} = 11$ hours; and $T_m = 0{\cdot}4$ hours (Table II).

### DISCUSSION

Previous methods by Morris have used the time between tumor transfers as an index for the differences in growth rates. This ranged between 0·6 months for the most rapidly growing tumor 3924A to 13 months for the slowest growing tumor 7794B. When increased size is computed from increases in the tumor dimensions measured along two axes, the change in tumor size for rapidly growing 3924A was calculated to be 8·4 cm. per month and for the slow growing 7794B to be 0·5 cm. per month. The time between tumor transfer and the rate of change in tumor size were plotted together (Morris and Wagner, 1968). The time between transfers was compared with the reciprocal of tumor size per month graphically. The correlation indicates the two methods of measurement of change in size and time between inoculation. The average time between tumor transfer of H-35tc$_2$ is 0·7 months (range 0·6–0·8 months). The change in the sum of the length and width of H-35tc$_2$ has been estimated by Morris and Wagner (1968) to be 7·0 cm. per month. It should be noted that two methods are in close agreement in that the total cell cycle times are calculated 22·2 hours and 22·9 hours, respectively.

The values for the different phases in the cycle for H-35tc$_2$ based on the computer program for producing optimum curves will be used in the following discussion. The calculated value of 22·2 hours for $T_c$ is similar to that found by Post and Hoffman (1965) in normal liver cells of 3 week old rats. The calculated value of 22·2 hours based on this method is longer than that found by Lesher (1967) in his studies of the intestinal crypt cells in the mouse. The $T_c$ for H-35tc$_2$ of 22·2 hours is also longer than the $T_c$ calculated by Bresciani (1968) to be 14–16 hours for a chemically induced rat hepatoma, and it is longer than the estimated $T_c$ of 13·8 hours for liver cells of 1 day old rats (Post and Hoffman, 1965).

The estimated value for the DNA synthetic period ($T_s$) of 6·6 hours is at the lower limits of the $T_s$ period of a number of tumors which were tabulated by Steel *et al.* (1966) and Steel (1968) and which range from 6–18 hours. However, it is close to the $T_s$ value of 8 hours for most mammalian cells (Steel and Bensted, 1965). The estimated value for $T_{g_2}$ of 4·2 hours is more than the usual for most mammalian cell lines.

The 1 hour thymidine labeling index was determined for Hepatomas 3924A, 9121 and H-35tc$_2$ in previous studies on the rates of thymidine incorporation into nuclear and mitochondrial DNA of these tumors between 1967–1968 (Chang, Morris and Looney, 1968). Two independent determinations for the 1 hour labeling index for Hepatoma 9121 were 13·9 and 13·4 per cent, respectively. The 1 hour labeling index for Hepatoma H-35tc$_2$ was 10·1 and 10·7 per cent for two experiments. Therefore, the per cent of labeled cells in the hepatomas is much greater than the normal liver ($0{\cdot}5 \pm 0{\cdot}04$ per cent) and the 13 hour regenerating liver ($0{\cdot}7 \pm 1$ per cent) before the onset of DNA synthesis. It is less than the $19{\cdot}1 \pm 2{\cdot}8$ per cent labeled liver cells at the peak of DNA synthesis in regenerating liver, 21 hours after partial hepatectomy (Looney and Mayo, 1969; Looney, Chang, and Banghart, 1967).

Steel and Bensted (1965) estimated the potential doubling times of tumors from the experimental data found on the labeling indices of various tumors and the time for DNA synthesis. A single equation is utilized to express this relationship based on the assumption that normal liver growth and the distribution of cell cycle times are invariant. The equation for the Potential Doubling Time is $[T] = T_S/L.I.$ where $T_S$ = time for DNA synthesis hours, L.I. = Labeling Index.

For cell populations in other than linear growth, one must take into account the fact that the probability of finding a cell in different parts of the cell cycle is not constant. In the extreme case of exponential growth, for instance, the phase distribution diagram is an exponential function. The effect of this on the equation is to introduce a constant of proportionality. $[T] = \lambda T_S/L.I.$ where $\lambda$ must be found from the shape of the phase distribution diagram. In the case of tumors, accurate values for $\lambda$ are generally not known, but for a wide range of tumor doubling times an assumed value of 0·80 appears sufficiently accurate within $\pm 10$ per cent. The estimated potential doubling time for H-35tc$_2$ based on this equation and using the 0·8 value for $\lambda$ is $T = 0{\cdot}8 \times 6{\cdot}6/12{\cdot}2 = 43$ hours.

Estimates of the proportion of proliferating cells in the tumor, which has been called the Growth Fraction by Mendelsohn (1962), can be made by determining the ratio of the per cent labeled cells to the per cent labeled mitoses. The Growth Fraction has also been estimated from the ratio of the experimentally determined 1 hour thymidine labeling index to the predicted 1 hour thymidine labeling index in the following manner (Steel, 1968): Predicted labeling index $= \lambda Ts/T_C$ where $T_S$ = duration of DNA synthesis and $T_C$ = duration of cell cycle, and $\lambda$ is the constant of proportionality. The predicted labeling index for H-35tc$_2$ is $0{\cdot}8 \times 6{\cdot}6/22{\cdot}2 = 23$ per cent.

$$\text{Growth Fraction} = \frac{\text{Experimentally determined 1 hour } H^3\text{TdR labeling index}}{\text{Predicted labeling index}}.$$

The Growth Fraction for Hepatoma H-35tc$_2$ is as follows:

$$\text{Growth Fraction} = 12{\cdot}2/23 = 53 \text{ per cent.}$$

It is interesting to note that the estimated potential doubling time is approximately twice the cell cycle time and that approximately one-half of the cell population is estimated to be proliferating cells.

In many instances, the tumor volume doubling time is used synonomously with cell population doubling time. Steel (1968) has enumerated different biological processes which can operate to modify this assumption which equates tumor volume with cell volume. Steel points out that the cell size distribution could change with time or there could be an accumulation of intercellular connective tissue, blood or cystic fluid. Either of these processes would obviously make tumor volume increase faster than the cell population so that the use of the volume doubling time would underestimate cell loss. Crude quantitative estimates of the relative amounts of connective tissue have been made on some of the Morris hepatomas. As much as one-third of the total tumor of H-35 might be connective tissue and incorporated blood vessels whereas tumors 9108 and 9121 both bear a close resemblance to normal liver which contains little connective tissue except in the portal triads.

Steel has also indicated that the effect of overt necrosis needs particular comment. Any region of dead tissue is probably in a dynamic state, being enlarged progressively by the addition of cells that die at its periphery but also being reduced to some extent by the process of resorption. The rate of such resorption is at present difficult to assess. If the necrotic portion of the tumor is constant over the period of determination of tumor growth rate, then the doubling time of the whole tumor is the same as its cellular part. However, if the necrotic portion is increasing, then the rate of cell loss will be underestimated.

The rate of cell loss from tumors may be a possible explanation for the apparent discrepancy between tumor growth rates and thymidine labeling indices (Steel, 1968; Denekamp, 1970) (see Table II). Some results have been presented that suggest that the cell loss increases with tumor size (Clifton and Yatvin, 1969). Therefore, one of the most significant findings from these hepatoma studies and from experiments in other laboratories is that cell loss appears to be an important and hitherto unrecognized factor in the net growth rate of tumors. Unpublished data from other institutions have shown that differing and probably erroneous conclusions may be drawn about the effects of the administration of various chemotherapeutic agents. This is because of an apparent failure to differentiate between the cytocidal and cytostatic effects of these agents, and to recognize the attending critical information to the problem of cell loss (Hofer *et al.*, 1969). Therefore, these findings may eventually alter our present concepts about the clinical management of cancer. Measurements of the actual volume doubling times will be necessary to determine if cell loss is contributing to the net tumor growth of Hepatoma H-35tc$_2$. These determinations are being made in accordance with the criteria established for the analysis of tumor growth curves by Dethlefsen, Prewitt and Mendelsohn (1968).

The authors would like to express their appreciation to Dr. Lyle Dethlefsen with regard to the problems of the growth curves, to Dr. Manabu Takahashi with regard to discussions on the PLM curves, and to Dr. Mortimer Mendelsohn for the number of discussions with regard to the overall problems of this initial study of cell kinetics and cell proliferation in these hepatomas. The authors are appreciative of the assistance of Professor L. F. Lamerton and his group in the Department of Biophysics, Royal Cancer Hospital, London, for the computer analysis of the data contained in this report which were from the program of Barrett. The technical assistance of Mrs. Oneida Mason and Mr. Harold Ragland is gratefully acknowledged. The technical assistance for preparation of the tumor-bearing rats by Mrs. C. M. Jackson and Mrs. Jean W. Lewis is also gratefully acknowledged.

The research reported herein was supported in part by U.S. Public Health Service Grants No. GM-10754, No. CA-10729, American Cancer Society Grant No. P-497, the Lilly Research Laboratories, and the Milheim Foundation for Cancer Research.

### REFERENCES

BARRETT, J. C.—(1966) *J. natn. Cancer Inst.*, **37**, 443.
BRESCIANI, F.—(1968) *Eur. J. Cancer*, **4**, 343.

CHANG, L. O., MORRIS, H. P. AND LOONEY, W. B.—(1968) *Br. J. Cancer*, **22**, 860.
CLIFTON, K. H. AND YATVIN, M. B.—(1969) *Proc. Am. Ass. Cancer Res.*, **10**, 14.
DENEKAMP, J.—(1970) *Cancer Res.*, **30**, 393.
DETHLEFSEN, L. A., PREWITT, J. M. S. and MENDELSOHN, M. L.—(1968) *J. natn. Cancer Inst.*, **40**, 389.
HOFER, K. G., PRENSKY, W., ROSENOFF, S. AND HUGHES, W. L.—(1969) *Nature, Lond.*, **221**, 576.
LESHER, S.—(1967) *Radiat. Res.*, **32**, 510.
LOONEY, W. B. and MAYO, A. A.—(1969) *Radiat. Res.*, **39**, 456.
LOONEY, W. B., CHANG, L. O. AND BANGHART, F. W.—(1967) *Proc. natn. Acad. Sci. U.S.A.*, **57**, 972.
MENDELSOHN, M. L.—(1962) *J. natn. Cancer Inst.*, **28**, 1015.
MORRIS, H. P.—(1965) *Adv. Cancer Res.*, **9**, 227.
MORRIS, H. P. AND WAGNER, B. P.—(1968) ‘ Methods in Cancer Research ’, edited by H. Busch. New York (Academic Press), Vol. IV, p. 125.
NOWELL, P. C., MORRIS, H. P. AND POTTER, V. R.—(1967) *Cancer Res.*, **27**, 1565.
POST, J. AND HOFFMAN, J.—(1964) *J. Cell Biol.*, **22**, 341.—(1965) *Expl Cell Res.*, **40**, 333.
POTTER, V. R.—(1967) *Cancer Bull., Houston*, **19**, 91.
STEEL, G. G.—(1968) *Cell Tissue Kinet.*, **1**, 193.
STEEL, G. G., ADAMS, K. AND BARRETT, J. C.—(1966) *Br. J. Cancer*, **20**, 784.
STEEL, G. G. AND BENSTED, J. P. M.—(1965) *Eur. J. Cancer*, **1**, 275.